# Supplementary material for: Abasy Atlas v2.2: The most comprehensive and up-to-date inventory of meta-curated, historical, bacterial regulatory networks, their completeness and system-level characterization
Source: Comput Struct Biotechnol J. 2020 May 16;18:1228–37. doi: 10.1016/j.csbj.2020.05.015 (PMC7283102; doi:10.1016/j.csbj.2020.05.015)
Supplement: Supplementary data 1 [file mmc1.pdf]

Supplementary information for

Abasy Atlas v2.2: The most comprehensive inventory of meta-curated, historical and up-to-date, bacterial regulatory networks, their completeness and system-level characterization

Juan M. Escorcia-Rodríguez[1], Andreas Tauch[2], and Julio A. Freyre-González[1,*]

[1]Regulatory Systems Biology Research Group, Laboratory of Systems and Synthetic Biology, Center for Genomics Sciences, Universidad Nacional Autónoma de México. Av. Universidad s/n, Col. Chamilpa, 62210. Cuernavaca, Morelos, México

[2]Centrum für Biotechnologie (CeBiTec). Universität Bielefeld, Universitätsstraße 27, 33615. Bielefeld, Germany

***Corresponding author:** jfreyre@ccg.unam.mx (JAFG)

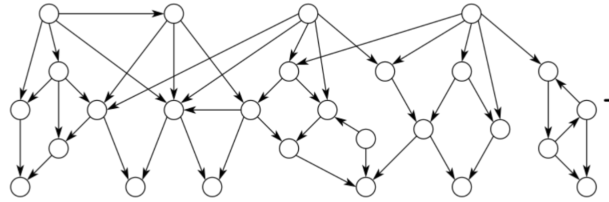

A regulatory network is represented as a directed graph. Each gene is a node, while arcs between them stand for regulatory interactions.

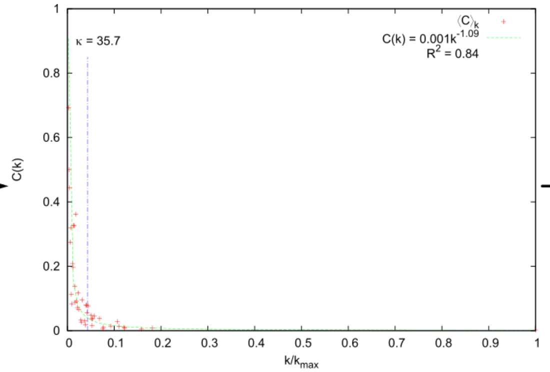

Global regulators are identified by the κ-value, which defines an equilibrium point between two contradictory behaviors: hubness (high out-degree, low clustering) and modularity (low out-degree, high clustering). First, the clustering coefficient distribution, $C(k)$, is computed. Then, given $C(k) = \gamma k^{-\alpha}$, the κ-value is calculated by using the formula $\kappa = \sqrt[\alpha+1]{\alpha\gamma} \cdot k_{\max}$.

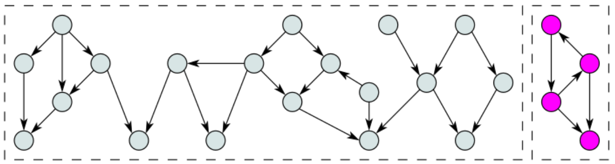

Identification of functional modules. Global regulators (connectivity > κ-value) and their links are removed from the network, thus naturally revealing the modules (isolated islands) and the basal machinery genes (single isolated nodes not encoding for regulators, not shown here for the sake of clarity).

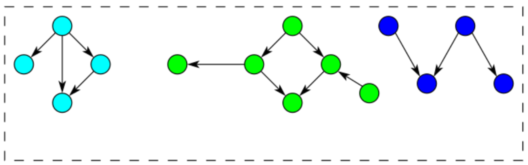

Identification of pre-submodules composing the megamodule. The megamodule is isolated and all the non-regulator-encoding genes and their links are removed, disaggregating it into isolated islands. Each island is identified as a pre-submodule.

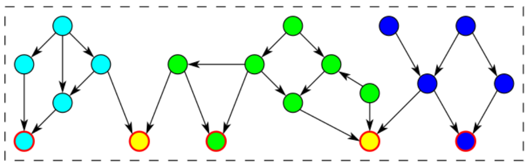

Identification of intermodular genes. All the removed non-regulators-encoding genes (red-outlined nodes) and their interactions are added back following a rule: if gene G is regulated by genes belonging to two or more submodules, then G is classified as intermodular (yellow nodes), else G is added to the same submodule than its regulators.

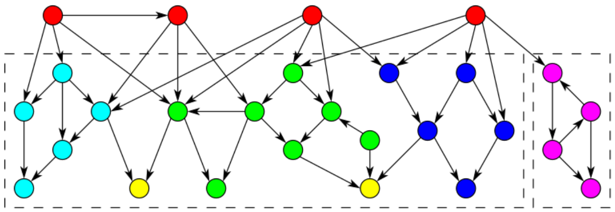

Finally, global regulators (red nodes) and their links are added back, thus reconstructing the original network but additionally revealing its hidden diamond-shaped three-layered architecture and its systems-level components: global regulators, modules, basal machinery and intermodular genes.

Supplementary Figure 1. Methodology of the natural decomposition approach (NDA).

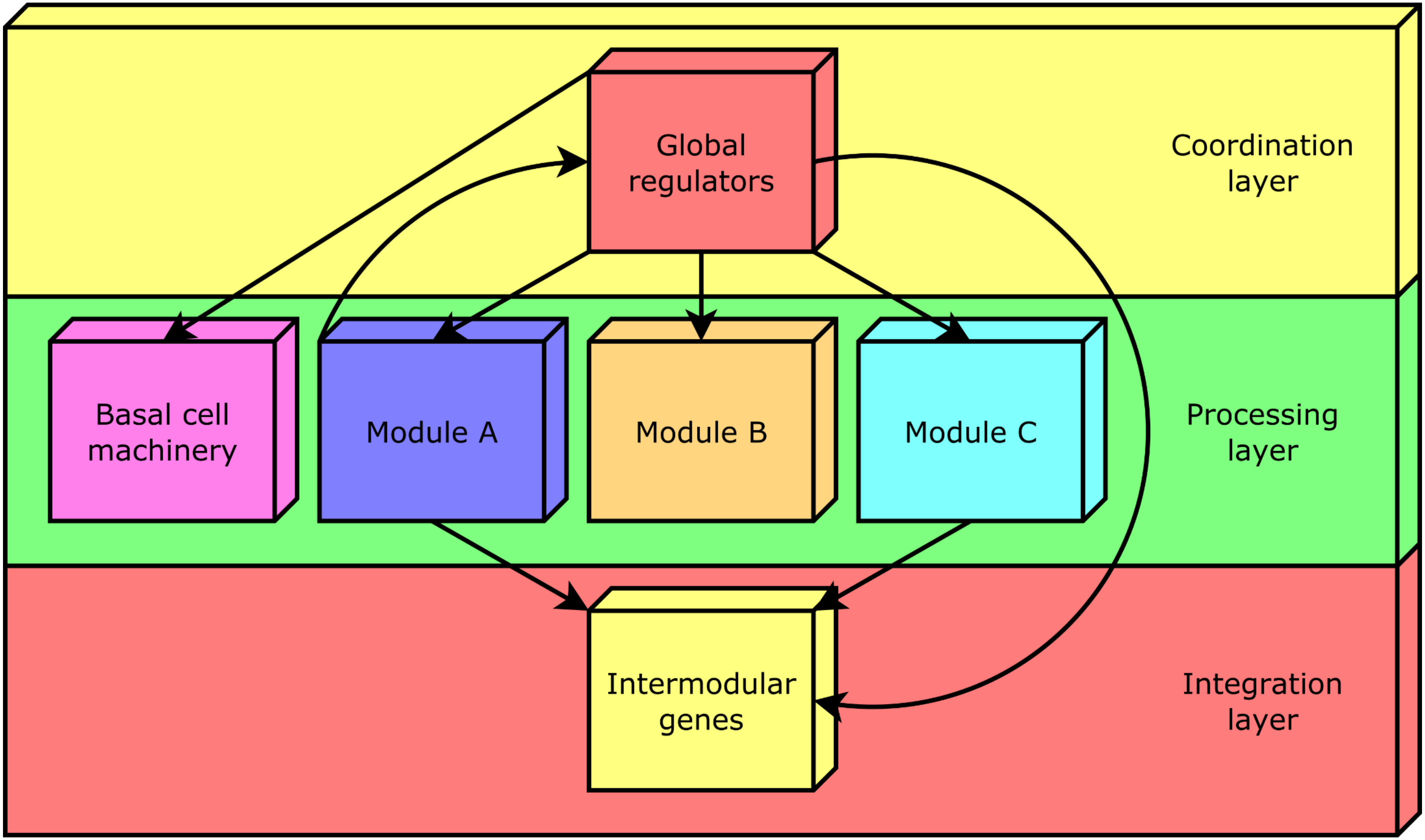

Supplementary Figure 2. The functional architecture unveiled by the NDA is a diamond-shaped, three-tier hierarchy, exhibiting some feedback between processing and coordination layers, which is shaped by four classes of system-level elements: global regulators, locally autonomous modules, basal machinery, and intermodular genes.

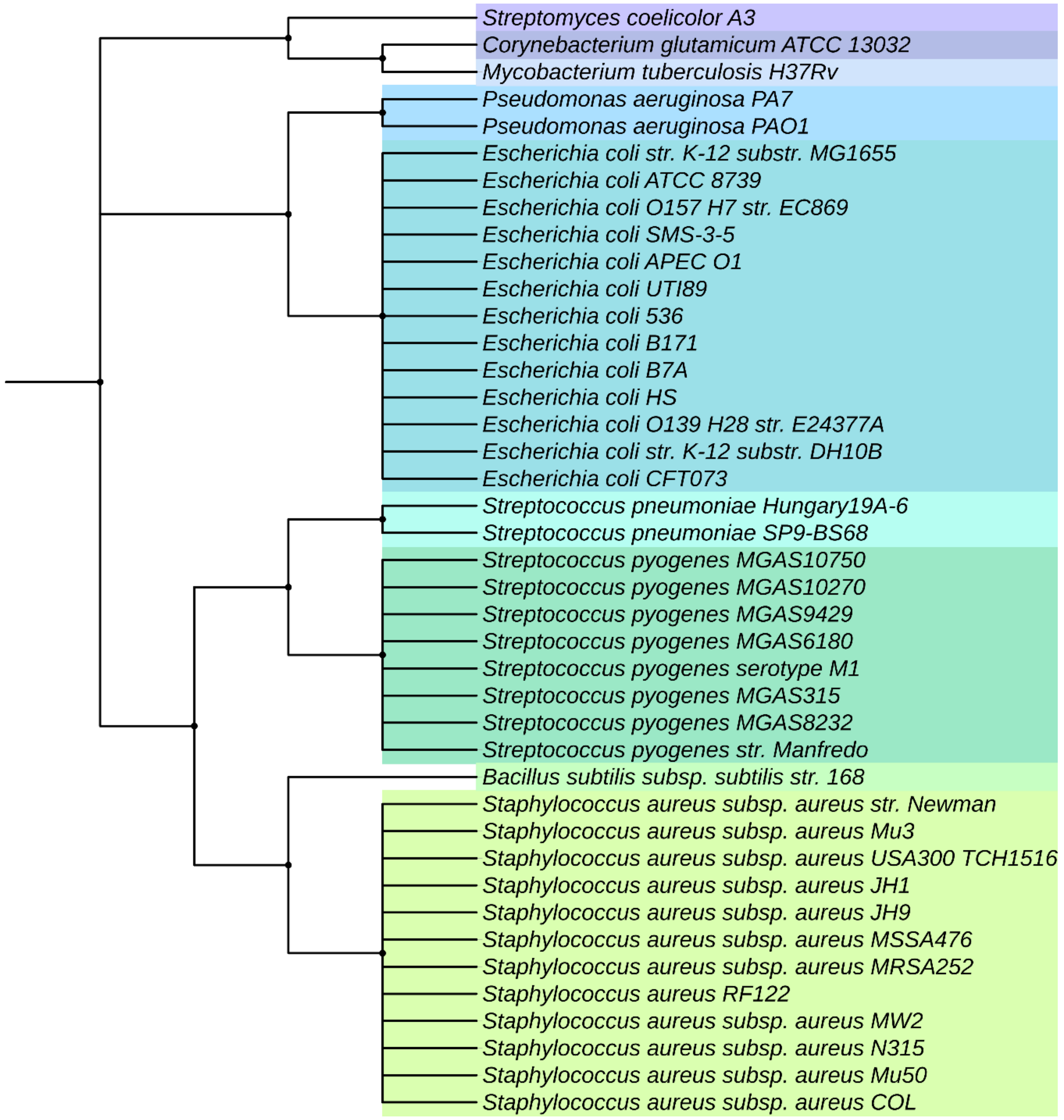

Supplementary Figure 3. Abasy integrates gene regulatory interactions for 9 species and 41 strains including model organisms.

DB abbreviation	PMID	Reference
**Bsub15**	26577401	[1]
**CRN12**	22080556	[2]
**Cglu17**	27829123	[3]
**DBTBS08**	17962296	[4]
**Mtuber11**	21818301	[5]
**Mtuber12**	22737072	[6]
**Mtuber15**	25581030	[7]
**Mtuber16**	27029515	[8]
**Paeru11**	22587778	[9]
**RDB01**	11125053	[10]
**RDB04**	14681419	[11]
**RDB06**	16381895	[12]
**RDB11**	21051347	[13]
**RDB13**	23203884	[14]
**RDB16**	26527724	[15]
**RDB18**	30395280	[16]
**RTB13**	23547897	[17]
**SW16**	26433225	[18]
**SW18**	29788229	[19]

Supplementary Table 1. Name abbreviation and year used as code for the source field in the network ID, and the PMID of the article.

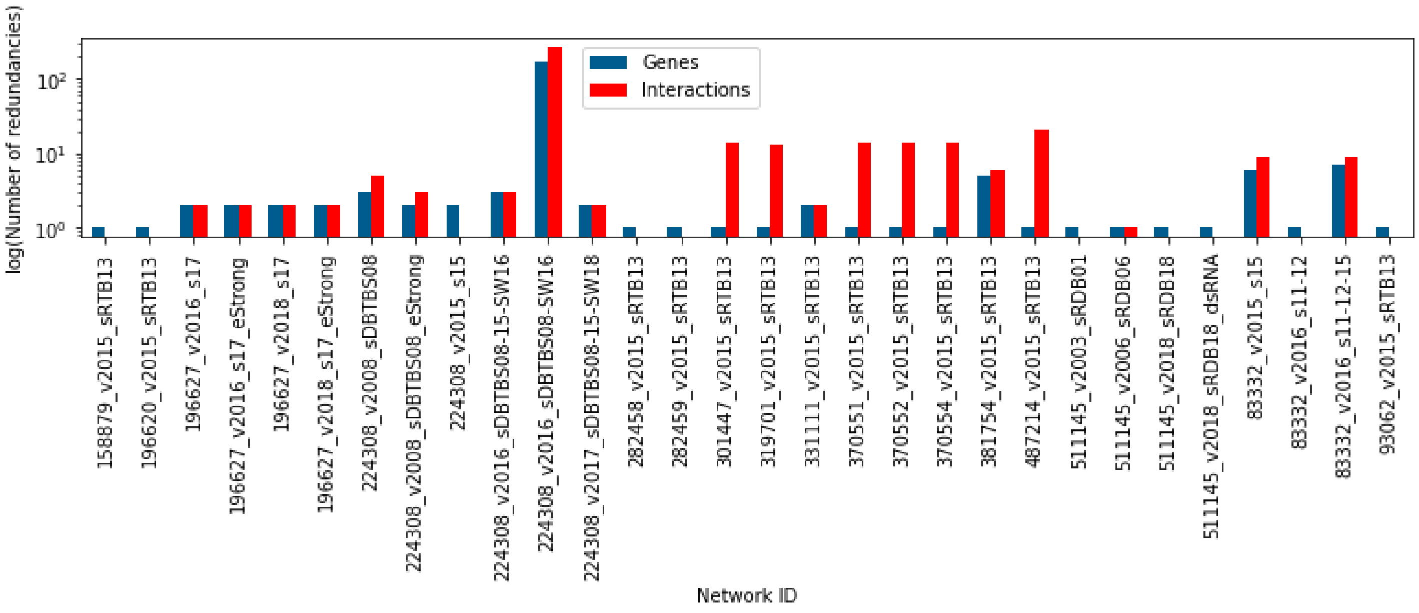

Supplementary Figure 4. Number of redundant genes and interactions removed due to the synonyms resolution in the meta-curated GRNs. A total of 223 nodes and 412 interactions are removed.

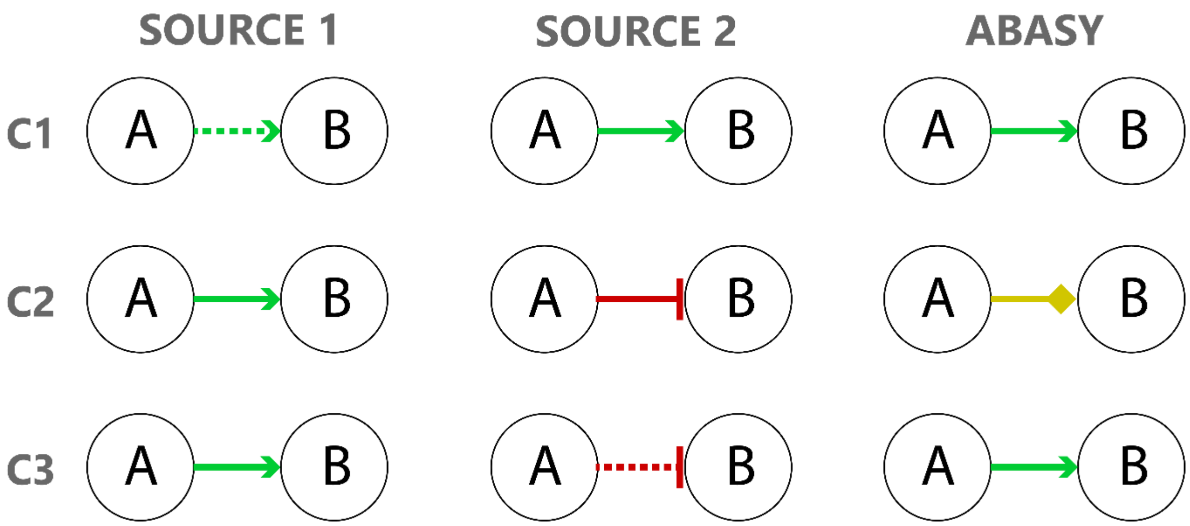

Supplementary Figure 5. Information preserved when consolidating regulatory interactions from different sources. **C1:** In case of same effect but different evidence level, the interaction shared and the "strong" evidence is conserved. **C2:** In case of different effects and the same evidence level, both effects are conserved in a single dual interaction to avoid redundancy. **C3:** In case of both attributes are different, only the "strong" interaction is conserved.

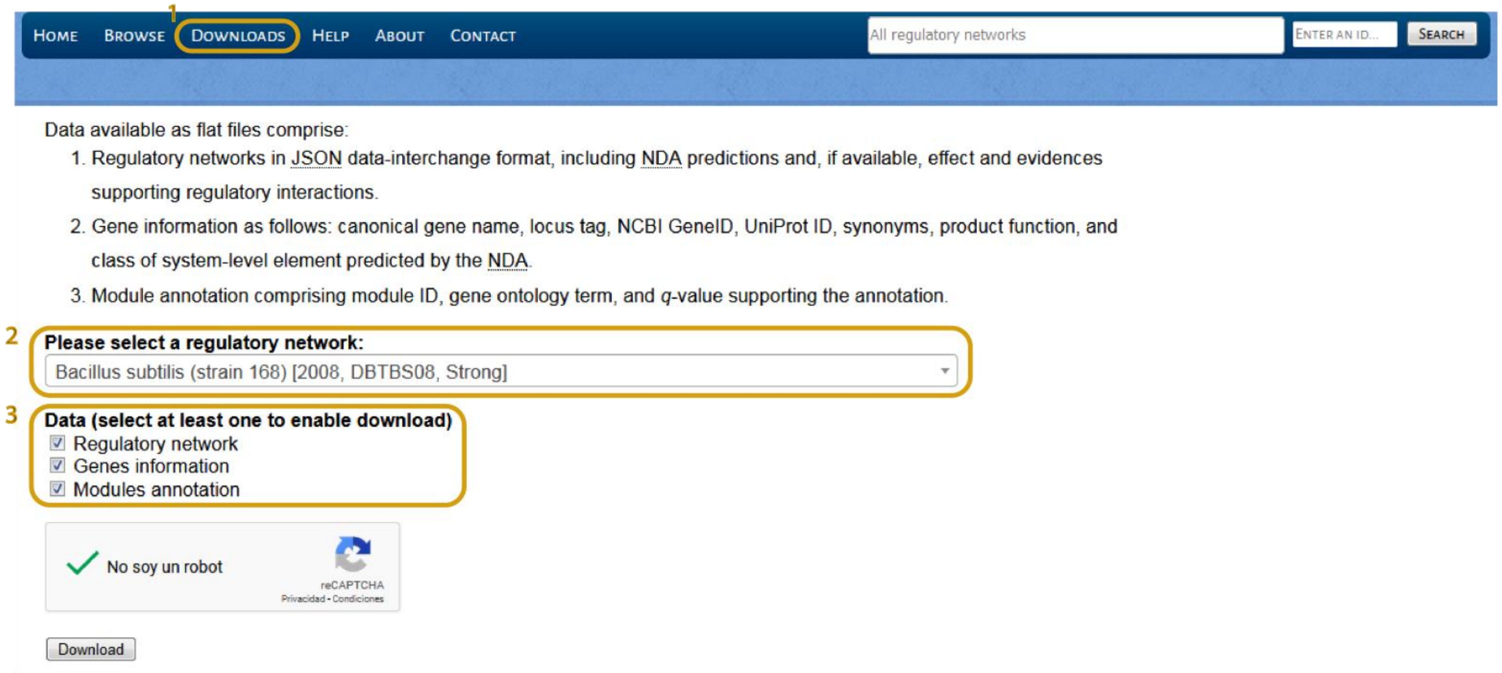

Supplementary Figure 6. Filtered GRNs to contain only "strong" interactions van be also downloaded through the "Downloads" page where you can select the GRN you want as well as the additional data such as gene information ad modules annotation.

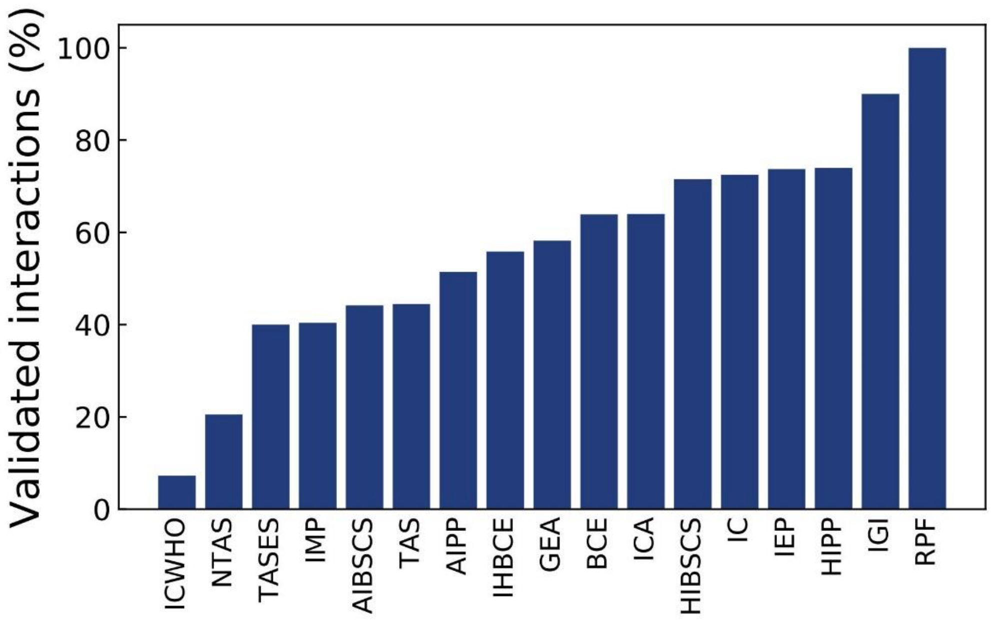

Supplementary Figure 7. Interactions inferred by a methodology classified as "weak" that has been validated with a "strong" experiment.

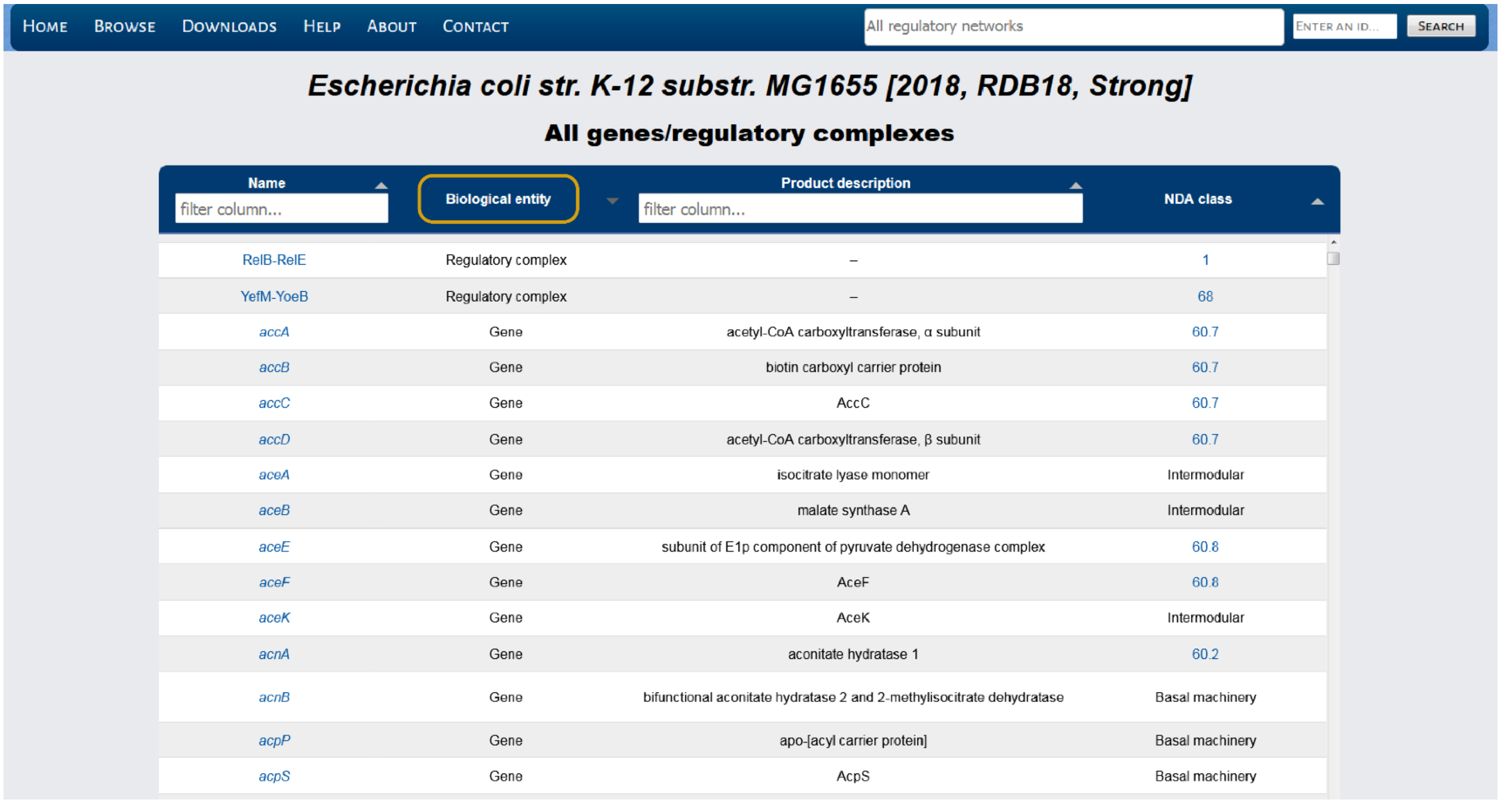

Name	Biological entity	Product description	NDA class
RelB-RelE	Regulatory complex	–	1
YefM-YoeB	Regulatory complex	–	68
accA	Gene	acetyl-CoA carboxyltransferase, α subunit	60.7
accB	Gene	biotin carboxyl carrier protein	60.7
accC	Gene	AccC	60.7
accD	Gene	acetyl-CoA carboxyltransferase, β subunit	60.7
aceA	Gene	isocitrate lyase monomer	Intermodular
aceB	Gene	malate synthase A	Intermodular
aceE	Gene	subunit of E1p component of pyruvate dehydrogenase complex	60.8
aceF	Gene	AceF	60.8
aceK	Gene	AceK	Intermodular
acnA	Gene	aconitate hydratase 1	60.2
acnB	Gene	bifunctional aconitate hydratase 2 and 2-methylisocitrate dehydratase	Basal machinery
acpP	Gene	apo-[acyl carrier protein]	Basal machinery
acpS	Gene	AcpS	Basal machinery

Supplementary Figure 8. Regulatory complexes can be identify using the "Biological entity" column and the Gene information file available at the "Downloads" page (see **Supplementary Figure 2)**. This makes possible to convert the GRNs to contain only gene-gene interactions when needed.

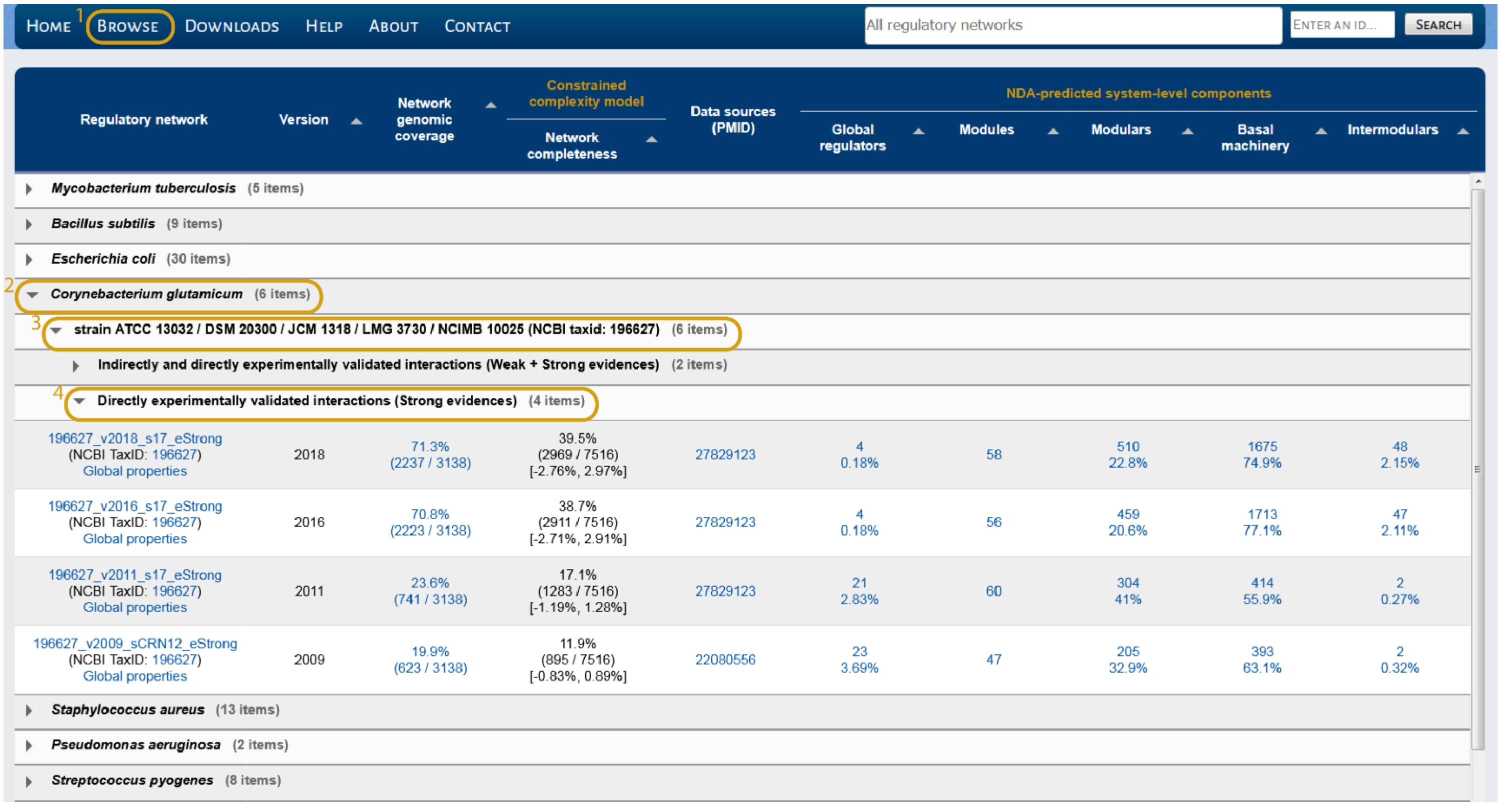

Supplementary Figure 9. From the "Browse" page you can identify the species and strain of interest, as well as the confidence level you need. Also, you will find additional information such as the GC and IC, data sources and fraction of the systems-level components predicted by the NDA. Clicking the hyperlink in the data sources PMID will open the PubMed page of the article for the data source.

HOME BROWSE DOWNLOADS HELP ABOUT CONTACT All regulatory networks ENTER AN ID... SEARCH

Mycobacterium tuberculosis (strain ATCC 25618 / H37Rv) [2018, 11-12-15-16, Weak + Strong]

Network global properties		
Regulators (k_{out} > 0)	–	179 (5.7%)
Structural genes (k_{out} = 0)	–	2955 (94.3%)
Undirected regulatory links	–	10119
Directed regulatory interactions	–	10147
Self-regulations	–	104 (58.1%)
Maximum out-connectivity	–	452 (14.4%)
Network density	–	0.00204
Weakly connected components	–	3
Genes in the giant component	–	3128 (99.8%)
Feedforward circuits	–	3994
Complex feedforward circuits	–	700
3-Feedback loops	–	63
Average path length	–	3.24
Network diameter	–	6
Average clustering coefficient	–	0.13008
$P(k)$	–	$0.772 \cdot k^{-1.97}$
R^2_{adj}	–	0.93

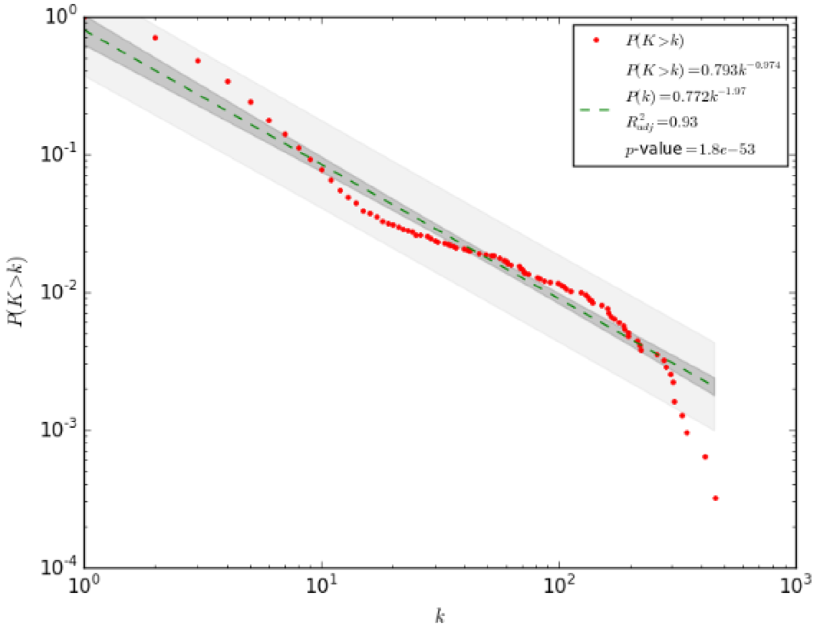

Supplementary Figure 10. Statistical and structural properties characterizing the GRN of interest.

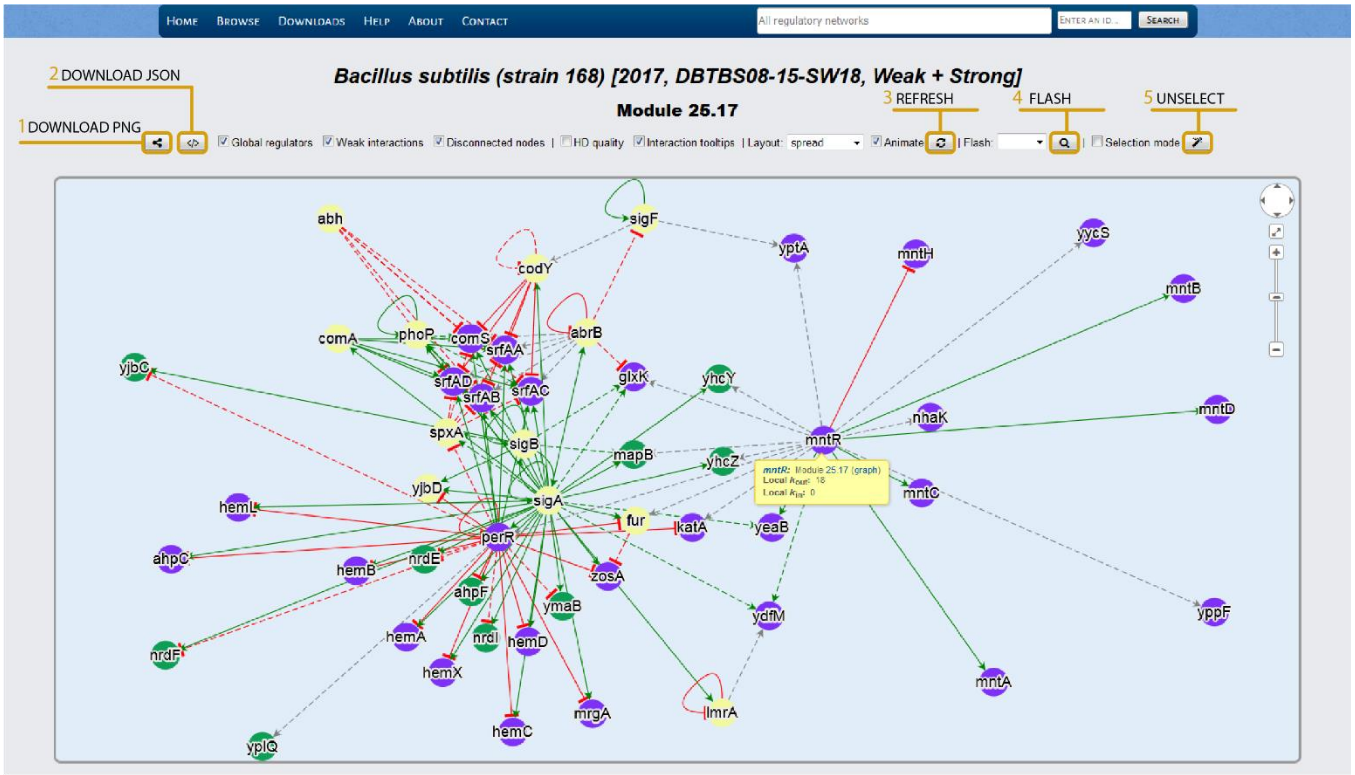

Supplementary Figure 11. On the interactive panel you can download the displayed network as an image in PNG format with transparent background (1). Also, you can download the JSON file (2) to import into Cytoscape and customize the style of the network. Check/uncheck the boxes to remove global regulators, “weak” interactions, disconnected genes, interaction tooltips and to display the network in high definition. One the desired boxes are checked, click the refresh button (3) to visualize the changes. Select one gene from the alphabetically sorted “Flash” list and click the flash button (4) to easily identify the gene of interest from the network. Click on the nodes to select them and drag the mouse to change the position of the selected nodes. To deselect all nodes, simply click the unselect button (5).

References

1. Arrieta-Ortiz, ML, Hafemeister, C, Bate, AR, Chu, T, Greenfield, A, et al. (2015) An experimentally supported model of the Bacillus subtilis global transcriptional regulatory network. Mol Syst Biol 11: 839.
2. Pauling, J, Rottger, R, Tauch, A, Azevedo, V, Baumbach, J (2012) CoryneRegNet 6.0--Updated database content, new analysis methods and novel features focusing on community demands. Nucleic Acids Res 40: D610-614.
3. Freyre-Gonzalez, JA, Tauch, A (2017) Functional architecture and global properties of the Corynebacterium glutamicum regulatory network: Novel insights from a dataset with a high genomic coverage. J Biotechnol 257: 199-210.
4. Sierro, N, Makita, Y, de Hoon, M, Nakai, K (2008) DBTBS: a database of transcriptional regulation in Bacillus subtilis containing upstream intergenic conservation information. Nucleic Acids Res 36: D93-96.
5. Sanz, J, Navarro, J, Arbues, A, Martin, C, Marijuan, PC, et al. (2011) The transcriptional regulatory network of Mycobacterium tuberculosis. PLoS One 6: e22178.
6. Rohde, KH, Veiga, DF, Caldwell, S, Balazsi, G, Russell, DG (2012) Linking the transcriptional profiles and the physiological states of Mycobacterium tuberculosis during an extended intracellular infection. PLoS Pathog 8: e1002769.
7. Minch, KJ, Rustad, TR, Peterson, EJ, Winkler, J, Reiss, DJ, et al. (2015) The DNA-binding network of Mycobacterium tuberculosis. Nat Commun 6: 5829.
8. Chauhan, R, Ravi, J, Datta, P, Chen, T, Schnappinger, D, et al. (2016) Reconstruction and topological characterization of the sigma factor regulatory network of Mycobacterium tuberculosis. Nat Commun 7: 11062.
9. Galan-Vasquez, E, Luna, B, Martinez-Antonio, A (2011) The Regulatory Network of Pseudomonas aeruginosa. Microb Inform Exp 1: 3.

10. Salgado, H, Santos-Zavaleta, A, Gama-Castro, S, Millan-Zarate, D, Diaz-Peredo, E, et al. (2001) RegulonDB (version 3.2): transcriptional regulation and operon organization in Escherichia coli K-12. Nucleic Acids Res 29: 72-74.
11. Salgado, H, Gama-Castro, S, Martinez-Antonio, A, Diaz-Peredo, E, Sanchez-Solano, F, et al. (2004) RegulonDB (version 4.0): transcriptional regulation, operon organization and growth conditions in Escherichia coli K-12. Nucleic Acids Res 32: D303-306.
12. Salgado, H, Gama-Castro, S, Peralta-Gil, M, Diaz-Peredo, E, Sanchez-Solano, F, et al. (2006) RegulonDB (version 5.0): Escherichia coli K-12 transcriptional regulatory network, operon organization, and growth conditions. Nucleic Acids Res 34: D394-397.
13. Gama-Castro, S, Salgado, H, Peralta-Gil, M, Santos-Zavaleta, A, Muniz-Rascado, L, et al. (2011) RegulonDB version 7.0: transcriptional regulation of Escherichia coli K-12 integrated within genetic sensory response units (Gensor Units). Nucleic Acids Res 39: D98-105.
14. Salgado, H, Peralta-Gil, M, Gama-Castro, S, Santos-Zavaleta, A, Muniz-Rascado, L, et al. (2013) RegulonDB v8.0: omics data sets, evolutionary conservation, regulatory phrases, cross-validated gold standards and more. Nucleic Acids Res 41: D203-213.
15. Gama-Castro, S, Salgado, H, Santos-Zavaleta, A, Ledezma-Tejeida, D, Muniz-Rascado, L, et al. (2016) RegulonDB version 9.0: high-level integration of gene regulation, coexpression, motif clustering and beyond. Nucleic Acids Res 44: D133-143.
16. Santos-Zavaleta, A, Salgado, H, Gama-Castro, S, Sanchez-Perez, M, Gomez-Romero, L, et al. (2019) RegulonDB v 10.5: tackling challenges to unify classic and high throughput knowledge of gene regulation in E. coli K-12. Nucleic Acids Res 47: D212-D220.
17. Cipriano, MJ, Novichkov, PN, Kazakov, AE, Rodionov, DA, Arkin, AP, et al. (2013) RegTransBase--a database of regulatory sequences and interactions based on literature: a resource for investigating transcriptional regulation in prokaryotes. BMC Genomics 14: 213.
18. Michna, RH, Zhu, B, Mader, U, Stulke, J (2016) SubtiWiki 2.0--an integrated database for the model organism Bacillus subtilis. Nucleic Acids Res 44: D654-662.
19. Zhu, B, Stulke, J (2018) SubtiWiki in 2018: from genes and proteins to functional network annotation of the model organism Bacillus subtilis. Nucleic Acids Res 46: D743-D748.
